# History of surgical approaches in orthopaedics

**DOI:** 10.1007/s00264-025-06541-0

**Published:** 2025-04-22

**Authors:** Jan Bartoníček, Ondřej Naňka

**Affiliations:** 1 Department of Orthopedics, First Faculty of Medicine, Charles University and the Central Military Hospital, Prague, Czech Republic; 2 Institute of Anatomy, First Faculty of Medicine, Charles University, Prague, Czech Republic

**Keywords:** History of orthopaedics, History of internal fixation, Surgical approaches

## Abstract

Surgical approaches in bone surgery have undergone a long evolution over more than 130 years. While a number of publications have been devoted to the history of internal fixation, surgical approaches have remained neglected from this perspective. The development of approaches in musculoskeletal surgery is inextricably linked to four personalities. Theodor Kocher, in 1892, pointed out that descriptions of surgical approaches must be an essential part of surgical textbooks of operative techniques; James Edwin Thompson, in 1918, formulated the basic requirements for the surgical approaches to the skeleton of limbs; Arnold Kirkpatrick Henry published the first textbook of surgical approaches in 1927 and presented the concept of internervous planes in 1945; in the same year, Toufick Nicola created the first comprehensive atlas of surgical approaches to bones and joints of limbs, the pelvis and spine.

## Introduction

A correct choice of the surgical approach and the ability to use it appropriately in practice are basic prerequisites for a successful operation. Surgical approaches in bone surgery have undergone a long evolution over more than 130 years. While a number of publications have been devoted to the history of internal fixation [1–4], surgical approaches have remained neglected from this perspective. The aim of this article is to highlight the milestones of their history.

### Basic prerequisites for development

The history of surgical approaches is intrinsically linked to the beginning of operative treatment of fractures. It took 50 years to establish the basic prerequisites for a successful development of internal fixation. The first of them was the introduction of anaesthesia in 1846, the second was antisepsis, later asepsis introduced gradually between 1867 and 1886, and the third and last one was discovery of X-rays in 1896 [4]. All these discoveries paved the way for gradual spreading of operative treatment of various fractures and non-unions.

### The first operations

The first descriptions of operative treatment of fractures appeared in case reports which, however, mentioned the surgical approach only marginally [5]. While in the case of subcutaneous fractures, e.g. of the patella, or olecranon, this can be understandable [6], in more deeply located fractures, such as those in the region of the forearm, it is rather surprising [7].

### T. Kocher 1892

The first to deal systematically with surgical approaches was a prominent Swiss surgeon Theodor Emil Kocher (1841–1917), the first winner of the Nobel Prize in Surgery. His name has been associated with a number of priorities [8]. Some of them are generally known (treatment of thyroid diseases, Kocher clamps, or the reduction maneuver for dislocations of the glenohumeral joint), others fell into oblivion. Few people know that Kocher was instrumental in introducing surgical gloves in Europe [9]. He also coined the now commonly used term metaphysis for the part of the long bone connecting the diaphysis and epiphysis [10].

In 1892, Kocher [11] published an outstanding textbook “*Chirurgische Operationslehre”* (*Textbook of operative surgery*), that was repeatedly published in both German [12] and English [13]. Already in the first edition, the description of many operations is supplemented with black-and-white drawings of skin incisions, and also, in some cases, with drawings showing individual phases of dissection. The quality and color of the illustrations improved over the following editions, and other surgical approaches were added. The high point was the 6th edition of the book of 1907 [12] containing a detailed description and color drawings of the approaches bearing Kocher’s name today– the Kocher approach to the elbow, or the Kocher-Langenbeck approach to the hip joint. Kocher described also an extensive surgical approach to the lateral aspect of the hindfoot, currently used in calcaneal fractures and called the lateral extensive approach.

### A. Lane and A. Lambotte 1905

William Arbuthnot Lane (1856–1943), a British surgeon from London, and Albin Lambotte (1866–1955), a Belgian surgeon from Antwerp, published between 1905 and 1914 the first textbooks on internal fixation [14–17]. While Lane [14, 15] did not address surgical approaches at all, Lambotte briefly mentioned several approaches to the hip joint, including a drawing of a skin incision, in the second edition [17].

### J.E. Thompson 1918

James Edwin Thompson (1863–1927), an English surgeon from Northwich, was in 1891 appointed professor of surgery at the University of Texas in Galveston. In cooperation with William Keiller (1861–1931), an anatomist from Scotland, they significantly raised the level of surgical education there, including practical training in the dissection room [18]. In 1918, Thompson published an extensive article, “*Anatomical methods of approach in operations on the long bones of the extremities”* [19], that can be considered the first manifesto of surgical approaches. An optimal approach should meet the following requirements:

- Easy access to the site of fracture, or focus of disease.
- Preservation of all nerves, both sensory and motor.
- Prevention of unnecessary injury to muscles.
- Preservation of the vascular supply. It must be borne in mind that any injury of the nutrient arteries of the bones must be avoided. Extensive denudation of the fractured ends of the bone should be avoided for the same reason.

In this article, Thompson described and depicted the topographic anatomy of the humerus, radius, ulna, femur, tibia and fibula, and presented surgical approaches to these bones. Currently, this article is cited only in the context of the posterolateral approach to the radial shaft, bearing Thompson´s name. The same name was given to the first and very successful vitallium hip prosthesis [20] designed by Thompson’s son Frederick Roeck Thompson (1907–1983).

### A.K. Henry 1927, 1945

Arnold Kirkpatrick Henry (1886–1962), an Irish surgeon and anatomist who worked for a long time in Cairo, published, in 1927, a thin book “*Exposures of long bones and other surgical methods”* [21]. It was the first textbook of surgical approaches. Henry briefly described there approaches to the humerus, radius, tibia and ulna. It is interesting that the book contains also a detailed description of the transsphenoidal approach to the pituitary gland.

In 1945, he published a more extensive textbook of surgical approaches “*Extensile exposure applied to limb surgery*” [22] which became world-famous. Here, Henry described the basic technical principles of surgical approaches, including the concept of so-called internervous planes, i.e. innervation interfaces of individual muscle groups, that are best suited for extensive approaches. The book dealt primarily with approaches to long bones of the upper and lower extremities, but not to their joints. The best known of the approaches described was the Henry approach to the radius, briefly presented as early as in 1926 [23] and still used successfully [24]. The textbook mentions also the tendinous intersection between the flexor hallucis longus and the flexor digitorum longus in the plantar aspect of the foot, currently known as *master knot of Henry* [25]. The second, expanded edition of “*Extensile exposure applied to limb surgery”* [26] was published in 1957 and has been reprinted in numerous editions. Nevertheless, it was not a typical atlas of surgical approaches, but rather an abundantly illustrated book presenting selected approaches, but omitting the limb joints, pelvis and spine.

In 1946, a thin book “*Anatomic Atlas of Orthopaedic Operation*” [27] was published by L.S. Michaelis from Botleys Park War Hospital in England. The book contains the basic approaches to the skeleton and limb joints, as well as approaches to the spine and the pelvis. The core of the book features simple black-and-white drawings supplemented by colored lines of skin incisions and a smaller number of colored, artistically more sophisticated, images. Depictions of approaches are often accompanied by a reference to their original descriptions, as they were taken from other authors, A.K. Henry in particular. Michaelis was also extensively involved in treatment of paraplegic patients.

### T. Nicola 1945

An American orthopedist Toufick Nicola (1894–1987) was the first to publish a true atlas of surgical approaches. Very little has been discovered about his life. It is evident from the literary sources that Nicola had been dealing over a long period with anterior dislocation of the shoulder [28] and described also his own technique of treatment. Cordasco [29] in 1938, mentioned Nicola in his article on the history of proximal femur fractures as the Chief of the Orthopedic Division of the Surgical Department in Mountainside Hospital in New Jersey. When Nicola in 1945 published his “*Atlas of surgical approaches to bones and joints”* [30], he was featured on the cover of the book as “formerly Chief of Clinic, the Hospital for the Ruptured and Crippled, New York“. It is possible that the book was written at the initiative of the U.S. Army, as the foreword was written by Norman T. Kirk, Major General, U.S. Army. Surgeon-General Kirk specialized in bone and joint surgery during World War 1 and was Surgeon General of the Army from 1943 to 1947, during the height of World War II.

The Atlas describes the basic approaches to the spine, pelvis and bones of the upper and lower extremities. The basis of the book consists of simple black-and-white, schematic, but very illustrative, drawings depicting the individual stages of the dissection. The Atlas was further expanded and the 1966 edition of the book [31] was translated into German by von Torklus and Türk in 1971 [32], presenting T. Nicola as the author. In subsequent German editions Nicola gradually disappeared as the author and in the 4th German edition, already in color, only von Torklus was identified as the author [33]. In a number of aspects, a striking resemblance can be found between von Torklus´ Atlas and the textbook of surgical approaches published by Brückner and Hinze [34] in 1980.

### AO/ASIF and others

The development of operative treatment in orthopaedics after World War II increased interest in the issue of surgical approaches. In 1953, Banks and Laufman published a comprehensive, well-illustrated book, “*An atlas of surgical exposures of the extremities”* [35], 2nd edition of which was published in 1987 [36].

A very important impetus for the study of surgical approaches was establishment of the AO Switzerland in 1958, later to become the AO/ASIF Foundation. Already the first AO-textbook of 1963 [37] mentioned some approaches, primarily to the hip, and presented simple drawings of skin incisions. In the AO-manuals published between 1969 and 1990 [38–40], mentions of surgical approaches and their illustrations were gradually, although unsystematically, increasing in numbers. The AO-textbook of *Surgical approaches for internal fixation* appeared as late as in 1984 [41]. This thin textbook contained only some approaches to the joints and bones of the upper and lower extremities. Despite very instructive drawings, however, it did not achieve greater popularity. In the same year (1984), Stanley Hoppenfeld and Piet de Boer published “*Surgical exposures in orthopaedics”* [42]. The book became very popular and was gradually improved and supplemented with additional approaches. In 2021, its 6th edition was published [43].

Rudolf Bauer, Fridun Kerschbaumer and Sepp Poisel from Innsbruck, in Austria, published, in 1986, an extensive, representative and splendidly illustrated textbook “*Operative Zugangswege in Orthopädie und Traumatologie”* [44], which one year later was also translated into English (*Operative approaches in orthopedic surgery and traumatology*) [45]. In 2019, the 5th German edition was published [46].

During the 1990s, there appeared other atlases [47, 48] and more recently also atlases devoted to only one anatomical region, i.e. the foot and ankle [49].

## Conclusion

The development of surgical approaches in musculoskeletal surgery is inextricably linked to four personalities. Theodor Kocher, in 1892, pointed out that descriptions of surgical approaches must be an essential part of surgical textbooks of operative techniques; James Edwin Thompson, in 1918, formulated the basic requirements for the surgical approaches to the skeleton of limbs; Arnold Kirkpatrick Henry published the first textbook of surgical approaches in 1927 and presented the concept of internervous planes in 1945; in the same year, Toufick Nicola created the first comprehensive atlas of surgical approaches to bones and joints of limbs, the pelvis and spine.

## Data Availability

No datasets were generated or analysed during the current study.

## References

[1] Peltier LF (1990) Fractures: a history and iconography of their treatment. Norman Publishing, San Francisco

[2] Colton CL (2009) The history of fracture treatment. In: Browner BD, Jupiter JB, Levine AM, Trafton PG, Ch K (eds) Skeletal trauma. Saunders, Philadelphia, pp 3–31

[3] Bartoníček J (2010) Early history of operative treatment of fractures. Arch Orthop Trauma Surg 130:1385–1396. 10.1007/s00402-010-1082-7 20217103 10.1007/s00402-010-1082-7

[4] Newman K (2024) Who´s who in trauma. Norwich, Swallowtail Print

[5] Annadale T (1875) Ununited fracture of the forearm, with deficiency of the Ulna, treated successfully by excision and the wire suture. Br Med J 1:38 10.1136/bmj.1.732.38 PMC2295391 20747718

[6] Bartoníček J, Rammelt S (2015) Early history of operative treatment of patellar fractures. Int Orthop 39(11):2303–2308. 10.1007/s00264-015-2768-9 25913262 10.1007/s00264-015-2768-9

[7] Hansmann H (1886) Eine neue Methode der Fixierung der Fragmente bei complicierten Fracturen. Verh Dtsch Ges Chir 15:134–137

[8] Bumbasirević MZ, Zagorac SG, Lesić AR (2013) Emil Theodor Kocher (1841–1917) -orthopaedic surgeon and the first surgeon Nobel prize winner. Acta Chir Iugosl 60(3):7–11. 10.2298/aci1303007b 24669574 10.2298/aci1303007b

[9] Hernigou P, Boceno A, Potage D (2023) Rubber gloves in orthopaedic surgery (part II): Cooke and Goodyear; Halsted and Caroline’s gloves of love; from cotton to rubber after Perthes’ experiments; double glove technique with urist. Int Orthop 47(4):1115–1123. 10.1007/s00264-022-05666-w 36565354 10.1007/s00264-022-05666-w

[10] Naňka O, Bartoníček J (2024) Terminology of the growing bone: A historical study. Clin Anat 37(7):761–768. 10.1002/ca.24176 38778675 10.1002/ca.24176

[11] Kocher T Chirurgische Operationslehre. 1. Auflage., Jena (1892) Fischer: https://archive.org/details/chirurgischeoper00koch/page/n3/mode/2 *up*

[12] Kocher T Chirurgische Operationslehre. 5. Auflage., Jena (1907) Fischer: https://archive.org/details/chirurgischeoper1907koch/mode/2 *up*

[13] Kocher T (1911) Textbook of operative surgery, 3rd edn. Macmillan, New York

[14] Lane WA (1905) The operative treatment of fractures. Medical publishing Co, London

[15] Lane WA (1914) The operative treatment of fractures, 2nd edn. Medical Publishing Co, London

[16] Lambotte A (1907) L’ intervention opératoire Dans les fractures récentes et anciennes envisagées particulièrement du point de vue de l’ostéosynthèse. Lambertin, Brussels

[17] Lambotte A (1913) Chirurgie opératoire des fractures. Masson, Paris

[18] Burns CR, Campbell HG (1999) The extraordinary influences of two British physicians on medical education and practice in Texas at the turn of the 20th century. Vesalius 5(2):79–84 11624232

[19] Thompson JE (1918) Anatomical methods of approach in operations on the long bones of the extremities. Ann Surg 68:309–329 17863988 10.1097/00000658-191809000-00012 PMC1427041

[20] Thompson FR (1954) Two and a half years’ experience with a vitallium intramedullary hip prosthesis. J Bone Joint Surg Am 36–A(3):489–502 13163080

[21] Henry AK (1927) Exposures of long bones and other surgical methods. Bristol, John Wright 1927

[22] Henry AK (1945) Extensile exposure applied to limb surgery. Edinburgh, Livingstone

[23] Henry AK (1926) Complete exposure of the radius. Br J Surg 13(51):506–508. 10.1002/BJS.1800135109

[24] Bartoníček J, Kozánek M, Jupiter JB (2014) History of operative treatment of forearm diaphyseal fractures. J Hand Surg Am. 39(2):335–342. 10.1016/j.jhsa. 2013. 06. 020 10.1016/j.jhsa.2013.06.020 24332651

[25] Beger O, Çalışır ES, Sevmez F, İnce R, Özdemir A, Keskinbora M (2022) Arnold Kirkpatrick Henry (1886–1962) and his eponym (Master knot of Henry): a narrative review. Surg Radiol Anat 44(1):157–168. 10.1007/s00276-021-02847-x 34611753 10.1007/s00276-021-02847-x

[26] Henry AK (1957) Extensile exposure applied to limb surgery, 2nd edn. Edinburgh, Churchill Livingstone

[27] Michaelis LS (1946) Anatomic atlas of orthopaedic operations. Heinemann, London

[28] Nicola T (1949) Acute anterior dislocation of the shoulder. J Bone Joint Surg Am 31–A(1):153–159 18106758

[29] Cordasco P (1938) Evolution of treatment of fracture of neck of femur. Arch Surg 37:871–927

[30] Nicola T (1945) Atlas of surgical approaches to bones and joints. Macmillan Company, New York

[31] Nicola T (1966) Atlas of orthopaedic exposures. Baltimore, Williams & Wilkins

[32] Nicola T (1971) Atlas operativer Zugangswege in der Orthopädie. Urban und Schwarzenberg, München

[33] von Torklus HD (1992) Atlas orthopädisch-chirurgischer Zugangswege. 4. Auflage. München, Urban und Schwarzenberg

[34] Brückner H, Hinze M (1980) Zuganswege in der Traumatologie. Leipzig, Barth

[35] Banks SW, Laufman H (1953) An atlas of surgical exposures of the extremities. Philadelphia, Saunders

[36] Banks SW, Laufman H (1987) An atlas of surgical exposures of the extremities. 2nd edit. Philadelphia, Saunders 1987

[37] Müller ME, Allgöwer M, Willeneger H (eds) (1963) Technik der operativen Frakturbehandlung. Springer, Berlin

[38] Müller ME, Allgöwer M, Willeneger H (eds) (1969) Manual der osteosynthese. Springer, Berlin

[39] Müller ME, Allgöwer M, Schneider R, Willeneger H (eds) (1977) Manual der osteosynthese. Springer, Berlin

[40] Müller ME, Allgöwer M, Willeneger H, Schneider R (eds) (1991) Manual of internal fixation. Springer, Berlin

[41] Ruedi T (1984) Surgical approaches for internal fixation. Springer, Berlin

[42] Hoppenfeld S, de Boer P (1984) Surgical exposures in orthopaedics. 1st edition. Philadelphia, JB Lippincott Company

[43] de Boer P, Buckley R, Hoppenfeld S (2021) Surgical exposures in orthopaedics: The Anatomic Approach. 6th edition. Philadelphia, Wolthers Kluwer

[44] Bauer R, Kerschbaumer F, Poisel S (1986) Operative Zugangswege in Orthopädie und Traumatologie. Thieme, Stuttgart

[45] Bauer R, Kerschbaumer F, Poisel S (1987) Operative approaches in orthopedic surgery and traumatology. Thieme, Stuttgart

[46] Kerschbaumer F, Weise K, Wirth CJ (Hrgs) (2019) Operative Zugangswege in Orthopädie und Traumatologie. Thieme, Stuttgart

[47] Colton CL, Hall AJ (eds) (1991) Atlas of orthopaedic surgical approaches. Butterworth Heinemann Ltd., Oxford

[48] Williams RP, Heckman JD (1997) Contemporary extensile exposures in orthopaedic surgery. Williams & Wilkins, Philadelphia

[49] de Boer P, Buckley R, Hoppenfeld S (2012) Surgical exposures in foot and ankle surgery. Wolters Kluwer-Lippincot Williams & Wilkins, Philadelphia

